# Hemodialysis professionals’ knowledge, attitudes, and practices toward venous thrombosis prevention: a multicenter survey

**DOI:** 10.1080/0886022X.2026.2707036

**Published:** 2026-09-14

**Authors:** Qian Mi, Xuejuan Zhuang, Lifang Zhou, Caixia Li, Mi Zhong, Shengzi Liu, Yina Zhao, Xia Fu

**Affiliations:** ^a^ School of Nursing, Guangdong Pharmaceutical University, Guangzhou, Guangdong, China; ^b^ The Eighth Affiliated Hospital, Sun Yat-Sen University, Shenzhen, Guangdong, China

**Keywords:** Hemodialysis professionals, venous thromboembolism, self-perceived knowledge-attitude-practice, VTE prevention, associated factors, cross-sectional study

## Abstract

Venous thromboembolism (VTE) is a serious complication in patients undergoing hemodialysis. This cross-sectional study investigated the self-perceived knowledge, attitude, and practice (KAP) regarding VTE prevention among hemodialysis professionals and explored the factors influencing their preventive practices. Hemodialysis professionals were recruited using convenience sampling, and data were collected *via* the Wenjuanxing platform using a general information questionnaire and a self-developed KAP questionnaire on VTE prevention. A total of 515 questionnaires were distributed, and 474 valid questionnaires were returned, yielding a valid response rate of 92.03%. The mean total KAP score for VTE prevention was 164.12 ± 27.74, with a standardized score of 71.36 ± 12.06, indicating a moderately high overall level. Self-perceived knowledge, attitude, and practice were correlated with each other (*r* = 0.389–0.424, *p* < 0.001). Multivariate stepwise linear regression analysis showed that age, hospital type, hospital level, number of daily dialysis shifts, implementation of VTE risk assessment, awareness of VTE-related guidelines, and training frequency were significant associated factors of the total KAP score, among which the implementation of VTE risk assessment was the most important factor (*p* < 0.05). These findings suggest that targeted training programs, strengthened clinical management mechanisms, and improved awareness and application of VTE risk assessment are needed to further enhance the standardization and effectiveness of VTE prevention in hemodialysis patients.

## KEY MESSAGES

1. What is known: Venous thromboembolism (VTE) is a serious complication in hemodialysis patients, but the knowledge, attitudes, and practices of hemodialysis professionals regarding VTE prevention remain understudied.
2. This study adds: Hemodialysis professionals in China have moderate overall KAP levels (standardized score 71.36 ± 12.06), with the lowest scores in self-perceived knowledge and the highest in attitudes; mandatory VTE risk assessment is the strongest predictor of better practice.
3. Potential impact: Implementing mandatory VTE risk assessment protocols, improving guideline awareness, and providing targeted, continuous training may effectively enhance VTE prevention in hemodialysis settings.

## 1. Introduction

Venous thromboembolism (VTE), which includes deep vein thrombosis (DVT) and pulmonary thromboembolism (PTE), represents the third most common vascular disorder globally, with its incidence continuing to rise [1]. In Europe and the United States, the annual incidence of DVT ranges from 53 to 162 per 100,000 population, while that of PTE ranges from 39 to 115 per 100,000 population [2]. Data from China between 2007 and 2016 showed that the incidence of DVT increased from 3.2 to 17.5 per 100,000 population, and that of PTE rose from 1.2 to 7.1 per 100,000 population, with in-hospital mortality rates comparable to those in Western countries and longer average hospital stays among affected patients [3]. VTE not only increases healthcare burdens but also constitutes a major cause of unexpected in-hospital death [4,5].

Maintenance hemodialysis (MHD), defined as renal replacement therapy administered regularly for more than three months to patients with end-stage renal disease, represents a cornerstone of clinical management for this population [6]. Advances in dialysis technology and demographic aging have led to a substantial increase in the number of MHD patients globally. In China alone, the MHD population exceeded 1.027 million by 2024 and continues to grow [7], presenting significant long-term clinical and public health challenges. Among these challenges, VTE poses a particularly serious threat to MHD patients. Due to chronic inflammation, endothelial injury, coagulation dysfunction, and frequent vascular access use, MHD patients face a significantly elevated VTE risk, including a pulmonary embolism risk approximately twice that of the general population [8–10]. Consequently, effective VTE prevention has become a critical component of quality management in MHD care.

However, the implementation of VTE preventive measures remains suboptimal. In China, for instance, the rate of standardized VTE prophylaxis among high-risk patients is reportedly low, highlighting a significant gap between guideline recommendations and real-world practice [11]. In recent years, enhancing the capability of healthcare professionals in VTE prevention has been recognized as a key strategy for improvement [12]. Accordingly, from 2021 to 2026, the Chinese National Health Commission consistently included the improvement of standardized VTE prophylaxis rates among its national healthcare quality and safety goals, elevating VTE prevention to a priority level of health administration [13]. While systemic and policy-level efforts are crucial, the role of frontline hemodialysis professionals – primarily nurses and nephrologists – is fundamental. Their self-perceived knowledge, attitudes, and practices (KAP) directly determine the quality of VTE risk assessment, preventive care delivery, and patient education at the point of care [14].

The KAP theory, originating from health behavior research, provides a suitable framework for this study. According to this theory, knowledge serves as the foundation, attitudes function as motivational drivers, and practices represent the behavioral outcome [15]. The model conceptualizes behavioral change as a sequential process: knowledge acquisition, attitude formation, and practice adoption. This framework has been widely validated in chronic disease management and preventive care [15,16]. However, systematic research on KAP regarding VTE prevention among hemodialysis professionals is lacking. Internationally, few studies have focused on this setting, and domestic research in China remains limited [16,17].

Therefore, this study aims to investigate the current state of self-perceived knowledge, attitudes, and practices regarding VTE prevention among hemodialysis staff and to analyze the factors influencing them, thereby providing evidence to support the improvement of VTE prevention training and the optimization of clinical practice.

## 2. Materials and methods

### 2.1. Study design and participants

This cross-sectional study was conducted from September 2025 to October 2025, focusing on hemodialysis professionals from hospitals of varying levels. This study was approved by the ethics committee of The Eighth Affiliated Hospital, Sun Yat-sen University (Approval Number: 202503301). The front page of the questionnaire explicitly articulated the objective of the study, the principle of data anonymity, and the voluntariness of participation. Thus, the act of submitting the questionnaire was considered verbal informed consent. Written informed consent was not obtained because the survey involved no more than minimal risk and anonymous data collection made written signatures impractical. This consent procedure was approved by the ethics committee.

Convenience sampling was used to select eligible hemodialysis healthcare professionals as the study subjects. Inclusion criteria: licensed physicians or registered nurses working in blood purification centers; informed consent and voluntary participation in the study. Exclusion criteria included: visiting physicians and nurses, residents, clinical interns; and individuals not on duty during the survey period, including those on external training, maternity leave, sick leave, or personal leave.

Based on the descriptive study sample size, the estimated sample size was 5–10 times the total number of questionnaire items, with an additional 10% margin to account for non-response [18]. The final questionnaire comprised 46 items, with the sample size calculated as: sample size = number of items × (5–10) ÷ (1-10%) = (255–511). A total of 515 questionnaires were distributed, with 515 returned, of which 474 were valid, yielding a valid response rate of 92.03%. Ultimately, 474 valid samples were included in the analysis.

### 2.2. General demographic information questionnaire

The questionnaire was developed based on a review of the literature and relevant materials. It included the following items: gender, age, highest educational attainment, professional title, province/city of the hospital, hospital type (public/private), teaching hospital status, hospital grade, years of experience in hemodialysis work, employment type, weekly working hours, number of dialysis machines in the center, daily dialysis shifts in the department, whether VTE risk assessment was conducted in the department, commonly used VTE risk assessment tools, awareness of VTE-related guidelines, sources of VTE-related self-perceived knowledge, number of VTE-related training sessions attended, and main factors influencing VTE prevention practices.

### 2.3. Self-perceived knowledge-attitude-practice questionnaire of hemodialysis professionals on prevention of venous thromboembolism

The questionnaire was initially developed by the researchers based on a review of the literature and group discussions. The preliminary items were then refined through two rounds of Delphi expert consultation involving a total of 20 experts in nephrology and hemodialysis (including both medical and nursing professionals), all of whom held senior professional titles, to finalize the item pool. Reliability and validity tests were conducted among hemodialysis professionals in Guangdong Province who met the inclusion and exclusion criteria. The final questionnaire consisted of three dimensions and 46 items. The self-perceived knowledge dimension comprised 19 items, scored on a 5-point Likert scale ranging from ‘completely unaware’ (1 point) to ‘mastered’ (5 points), with a total possible score of 19–95. The attitude dimension included 16 items, also measured on a 5-point Likert scale from ‘strongly disagree’ (1 point) to ‘strongly agree’ (5 points), yielding a total score of 16–80. The practice dimension contained 11 items, rated on a 5-point Likert scale from ‘never’ (1 point) to ‘always’ (5 points), with a total score ranging from 11 to 55. The overall scale score ranged from 46 to 230, with higher scores indicating better self-perceived knowledge, more positive attitudes, and more appropriate practices regarding VTE prevention among hemodialysis healthcare workers. The item-level content validity index (I-CVI) ranged from 0.800 to 1.000, and the scale-level content validity index (S-CVI) was 0.899. The questionnaire demonstrated a Cronbach’s alpha coefficient of 0.977 and a split-half reliability of 0.883. The Cronbach’s alpha coefficients for the self-perceived knowledge, attitude, and practice dimensions were 0.983, 0.980, and 0.936, respectively. Exploratory factor analysis (EFA) was conducted with a sample of 327 participants. The Kaiser-Meyer-Olkin (KMO) value was 0.964, and Bartlett’s test of sphericity was significant (*χ^2^*= 19677.685, *p* < 0.001). Four factors were extracted, explaining 73.837% of the total variance. Overall, the questionnaire showed good reliability and validity. The full questionnaire is provided as Supplementary Material.

To meet the research objectives, standardized scores were calculated to assess the KAP level of hemodialysis professionals regarding VTE prevention. The specific calculation method was as follows: standardized score = (actual scale score/total possible score of the scale) × 100. Scores below 60 were defined as ‘poor’, scores between 60 and 80 as ‘moderate’, and scores of 80 or above as ‘good’ [19].

### 2.4. Data collection

Data for this study were collected through the Wenjuanxing online survey platform. The questionnaire was distributed to eligible participants with the assistance of the Chairperson of the Hemodialysis Nursing Committee of the Guangdong Nursing Association. All items in the questionnaire were set as required fields, and the same IP address was restricted to submit only once to control for duplicate responses. Responses that showed high similarity or internal inconsistency, or that were completed in less than 2 min, were considered invalid and excluded from analysis.

### 2.5. Analysis of data

Data analysis was performed using SPSS version 27.0. Descriptive statistics [means ± SD for normal data; medians (IQR) for non-normal data; frequencies (%) for categorical data] were calculated. Group comparisons used t-tests (normal, 2 groups), Wilcoxon-Mann–Whitney tests (non-normal, 2 groups), ANOVA (normal, ≥3 groups), and Kruskal-Wallis tests (non-normal, ≥3 groups). Correlations were assessed using Pearson (normal) or Spearman (non-normal) coefficients. Variables with potential associations were first screened through univariate analysis (independent t-tests for binary variables and ANOVA for multi-level categorical variables). Variables achieving a significance level of *p* < 0.05 in univariate analyses were subsequently entered into the multiple linear regression model to identify independent predictors of self-perceived knowledge, attitudes, and practices (α_in_=0.05_,_α_out_=0.10). Before regression analysis, we checked the assumptions of linearity (scatter plots), normality (Q-Q plots), and multicollinearity (VIF < 5). Statistical significance was set at *p* < 0.05.

## 3. Results

### 3.1. Basic information of the respondents

A total of 515 questionnaires were distributed, and 474 valid responses were obtained, yielding a valid response rate of 92.03%. The sample comprised 444 (93.67%) females and 30 (6.33%) males.This is close to the 2024 statistics from the National Health Commission, which show that 96.2% of nurses nationwide are female [20]. A total of 292 participants (61.60%) had fewer than 5 years of hemodialysis work experience. Regarding education, 238 respondents (50.21%) held a junior college diploma, and in terms of professional rank, 319 (67.30%) held junior-level titles. Detailed characteristics are presented in Table 1.

**Table 1.** Demographic characteristics and comparison of KAP scores among hemodialysis professionals (*N* = 474).

Characteristics	*N (*%)	Self-perceived knowledge ^a^	Attitude ^b^	Practice ^c^	Total score
Gender					
Male	30(6.33)	56.40 ± 15.70	66.87 ± 8.63	37.67 ± 9.38	160.93 ± 26.68
Female	444(93.67)	59.58 ± 16.58	65.78 ± 9.44	38.98 ± 9.22	164.34 ± 27.83
	*t*	1.021	−0.614	0.753	0.651
	*P*	0.308	0.540	0.452	0.516
Age (years)					
≤25	125(26.37)	58.03 ± 15.31	65.62 ± 7.51	39.27 ± 8.34	162.93 ± 22.70
26 ∼ 35	250(52,74)	56.71 ± 15.26	64.64 ± 9.96	38.11 ± 9.60	159.46 ± 27.15
36 ∼ 45	67(14.14)	66.78 ± 19.42	68.67 ± 10.19	39.70 ± 9.91	175.15 ± 33.63
≥46	32(6.75)	70.03 ± 16.08	70.25 ± 7.13	41.88 ± 7.33	182.16 ± 23.99
	*F*	12.149	5.953	1.973	11.138
	*P*	<0.001***	<0.001***	0.117	<0.001***
Education Level					
Junior college	238(50.21)	55.46 ± 14.66	64.56 ± 9.19	39.31 ± 8.62	159.33 ± 24.07
Bachelor’s Degree	225(47.47)	62.86 ± 17.50	67.06 ± 9.36	38.32 ± 9.90	168.24 ± 30.46
Master’s Degree and above	11(2.32)	73.18 ± 10.97	68.82 ± 10.93	41.64 ± 6.79	183.64 ± 23.26
	*F*	16.53	4.738	1.154	9.057
	*P*	<0.001***	0.009**	0.316	<0.001***
Professional Title					
Primary	319(67.30)	57.69 ± 15.59	65.15 ± 8.99	39.04 ± 9.04	161.88 ± 25.72
Intermediate	120(25.32)	61.03 ± 17.47	66.59 ± 10.46	37.89 ± 10.03	165.52 ± 31.06
Associate Senior	31(6.54)	68.00 ± 18.26	69.35 ± 6.95	40.03 ± 6.80	177.39 ± 26.93
Senior (Full)	4(0.84)	78.00 ± 15.51	72.00 ± 16.00	48.75 ± 10.53	198.75 ± 40.97
	*F*	6.212	2.888	2.195	5.382
	*P*	<0.001***	0.035*	0.088	0.001**
Hospital type					
Public	149(31.43)	68.60 ± 16.85	68.39 ± 9.44	41.20 ± 8.80	178.19 ± 29.15
Private	325(68.57)	55.15 ± 14.57	64.68 ± 9.14	37.84 ± 9.23	157.67 ± 24.55
	*t*	8.409	4.008	3.737	7.465
	*P*	<0.001***	<0.001***	<0.001***	<0.001***
Teaching hospital					
Yes	177(37.34)	65.73 ± 17.00	67.68 ± 10.78	40.62 ± 9.06	174.03 ± 30.14
No	297(62.66)	55.60 ± 15.04	64.75 ± 8.27	37.87 ± 9.18	158.22 ± 24.42
	*t*	6.551	3.110	3.167	5.919
	*P*	<0.001***	<0.001***	0.002**	<0.001***
Hospital level					
Tertiary hospital	137(28.91)	68.75 ± 17.01	68.71 ± 9.38	41.06 ± 9.41	178.52 ± 29.57
Secondary hospital	35(7.38)	57.83 ± 16.97	65.43 ± 12.91	37.37 ± 8.29	160.63 ± 30.18
Primary hospital	13(2.74)	50.69 ± 13.91	59.92 ± 6.46	36.15 ± 8.53	146.77 ± 17.87
Private hemodialysis center	289(60.97)	55.52 ± 14.48	64.81 ± 8.67	38.18 ± 9.13	158.51 ± 24.19
	*F*	24.381	7.463	3.868	20.349
	*P*	<0.001***	<0.001***	0.009**	<0.001***
Experience in Hemodialysis (Years)					
≤5	292(61.60)	57.22 ± 15.73	64.55 ± 9.77	39.04 ± 9.11	160.81 ± 26.69
6 ∼ 10	111(23.42)	61.25 ± 16.75	67.28 ± 7.05	38.36 ± 9.72	166.89 ± 27.70
11 ∼ 15	37(7.81)	62.00 ± 18.30	67.76 ± 10.79	36.46 ± 9.12	166.22 ± 30.71
≥16	34(7.17)	69.00 ± 16.65	70.24 ± 9.12	42.06 ± 8.00	181.29 ± 27.11
	*F*	6.505	5.881	2.362	6.38
	*P*	<0.001***	<0.001***	0.071	<0.001***
Employment Type					
Regular Staff	45(9.50)	70.73 ± 16.39	70.42 ± 7.91	41.40 ± 7.76	182.56 ± 26.59
Contract Staff	415(87.55)	58.21 ± 16.16	65.49 ± 9.49	38.67 ± 9.32	162.37 ± 27.37
Staff under Personnel Agency	3(0.63)	64.00 ± 18.52	58.67 ± 9.24	40.33 ± 6.43	163.00 ± 28.48
Dispatched Staff	11(2.32)	56.00 ± 14.38	62.55 ± 4.55	36.55 ± 10.69	155.09 ± 19.49
	*F*	8.363	4.896	1.461	7.865
	*P*	<0.001***	0.002**	0.227	<0.001***
Weekly working hours (hours)					
≤35	23(4.85)	57.74 ± 18.14	63.91 ± 8.86	40.04 ± 9.60	161.70 ± 31.23
36 ∼ 40	147(31.01)	61.70 ± 16.95	65.90 ± 9.74	39.78 ± 9.09	167.39 ± 29.18
>40	304(64.14)	58.38 ± 16.14	65.97 ± 9.26	38.38 ± 9.25	162.73 ± 26.70
	*F*	2.123	0.515	1.338	1.492
	*P*	0.121	0.598	0.263	0.226
Number of dialysis machines in the center					
≤20	38(8.02)	62.53 ± 18.38	66.05 ± 8.87	39.05 ± 8.49	167.63 ± 29.26
21 ∼ 40	186(39.23)	57.95 ± 16.24	66.09 ± 8.81	37.33 ± 9.56	161.37 ± 27.35
41 ∼ 60	152(32.07)	56.01 ± 15.34	64.55 ± 9.99	39.34 ± 9.08	159.89 ± 26.25
>60	98(20.68)	66.12 ± 16.21	67.33 ± 9.55	41.11 ± 8.65	174.56 ± 27.76
	*F*	8.894	1.843	3.87	6.862
	*P*	<0.001***	0.138	0.009**	<0.001***
Daily dialysis shifts					
1	4(0.84)	54.25 ± 15.84	64.50 ± 1.00	43.50 ± 4.04	162.25 ± 20.07
2	177(37.34)	55.97 ± 14.99	64.73 ± 8.39	37.94 ± 9.65	158.64 ± 25.96
2 ∼ 3	141(29.74)	59.16 ± 15.94	65.35 ± 11.12	37.35 ± 9.08	161.85 ± 28.51
3	108(22.79)	59.59 ± 17.20	66.82 ± 7.62	40.31 ± 7.99	166.72 ± 24.40
3 ∼ 4	44(9.29)	73.77 ± 15.59	69.68 ± 10.48	43.80 ± 9.14	187.25 ± 29.91
	*F*	11.219	2.924	5.665	10.651
	*P*	<0.001***	0.021*	<0.001***	<0.001***
Conduct VTE risk assessment					
Yes, assessment is mandatory	131(27.64)	63.18 ± 16.32	67.43 ± 9.88	42.89 ± 8.21	173.50 ± 26.91
Yes, assessment is implemented but not mandatory	212(44.72)	57.51 ± 15.29	64.50 ± 9.09	38.89 ± 8.18	160.91 ± 25.48
No	131(27.64)	58.61 ± 18.11	66.45 ± 9.11	34.90 ± 10.07	159.96 ± 29.99
	*F*	5.03	4.373	27.332	10.805
	*P*	0.007**	0.013*	<0.001***	<0.001***
Awareness of VTE-related guidelines					
Very familiar	18(3.80)	68.78 ± 11.77	67.39 ± 6.28	45.00 ± 7.62	181.17 ± 18.45
Somewhat familiar	107(22.57)	66.17 ± 16.75	67.63 ± 9.47	43.22 ± 7.17	177.02 ± 26.60
Moderately familiar	236(49.79)	59.32 ± 15.89	65.40 ± 9.13	38.97 ± 8.35	163.69 ± 25.57
Slightly familiar	104(21.94)	52.01 ± 14.97	65.27 ± 10.22	33.70 ± 10.13	150.98 ± 27.74
Not familiar at all	9(1.90)	46.67 ± 11.68	60.11 ± 6.88	33.33 ± 11.58	140.11 ± 23.00
	*F*	13.816	2.178	19.592	17.049
	*P*	<0.001***	0.070	<0.001***	<0.001***
Number of VTE-related training sessions attended in the past year					
0	191(40.30)	54.64 ± 15.95	65.48 ± 9.83	36.69 ± 10.12	156.81 ± 27.73
1∼2	241(50.84)	60.76 ± 15.58	65.64 ± 9.26	39.37 ± 8.19	165.78 ± 25.71
3∼4	30(6.33)	74.53 ± 16.14	68.53 ± 7.91	46.70 ± 4.63	189.77 ± 24.21
≥5	12(2.53)	69.17 ± 14.59	69.17 ± 6.82	44.92 ± 8.69	183.25 ± 25.92
	*F*	17.195	1.461	13.767	16.665
	*P*	<0.001***	0.224	<0.001***	<0.001***

Note: Data are presented as n (%) or Mean ± SD.

KAP, self-perceived knowledge, attitude, and practice.

^a^self-perceived knowledge: 19 Likert-scale items (1–5 points per item; total range: 19–95).

^b^Attitude: 16 Likert-scale items (1–5 points per item; total range: 16–80).

^c^Practice: 11 Likert-scale items (1–5 points per item; total range: 11–55).

*p < 0.05, **p < 0.01, ***p < 0.001

The total scores of self-perceived knowledge, attitude, and practice of hemodialysis professionals in VTE prevention ranged from 46 to 230 points. The actual scores of self-perceived knowledge, attitude, and practice in the survey were 164.12 ± 27.74 points, with a standard score of 71.36 ± 12.06 points, indicating an overall moderate level. Among the three dimensions, the actual score of self-perceived knowledge was 59.38 ± 16.53 points, with a standard score of 62.51 ± 17.40 points, also at a moderate level. The actual score of attitude was 65.85 ± 9.39 points, with a standard score of 82.31 ± 11.73 points, higher than that of self-perceived knowledge. The actual score of practice was 38.89 ± 9.22 points, with a standard score of 70.71 ± 16.76 points, lower than that of attitude but higher than that of self-perceived knowledge. These results indicate that hemodialysis professionals possess moderate levels of self-perceived knowledge, positive attitudes, and average practice levels in VTE prevention.

Univariate analysis (including t-tests and one-way ANOVA) revealed that, except for gender and weekly working hours, demographic factors such as age, education level, professional title, hospital type, and hospital level were significantly associated with KAP scores (Table 1). The results demonstrated that, apart from gender and weekly working hours, factors such as age, education level, professional title, hospital type, and hospital level were significantly correlated with the KAP scores.

### 3.2. Correlations among self-perceived knowledge, attitude, and practice regarding VTE prevention in hemodialysis professionals

Pearson correlation analysis was performed to examine the relationships among self-perceived knowledge, attitude, and practice regarding VTE prevention in hemodialysis professionals. The results indicated a significant correlation between self-perceived knowledge and attitude (*r* = 0.408, *p* < 0.001), between self-perceived knowledge and practice (*r* = 0.424, *p* < 0.001), and between attitude and practice (*r* = 0.389, *p* < 0.001). Overall, the correlations among these three dimensions range from 0.389 to 0.424, indicating a moderate correlation. All correlations are statistically significant. Details are shown in Table 2.

**Table 2.** Correlation of self-perceived knowledge, attitude, and practice scores (*N* = 474).

	Self-perceived knowledge	Attitude	Practice
self-perceived knowledge	1.000		
Attitude	0.408**	1.000	
Practice	0.424**	0.389**	1.000
Total	0.875**	0.711**	0.717**

Note: **p < 0.001

### 3.3. Multivariate analysis of KAP on VTE prevention among hemodialysis professionals

The three dimensions of KAP regarding VTE prevention among hemodialysis professionals were used as dependent variables. Univariate analysis of variance revealed that statistically significant demographic characteristics were selected as independent variables (*p* < 0.05). To ensure the scientific rigor and standardization of the regression analysis, the total score and dimension scores were treated as factor variables, and stepwise multiple linear regression was used to construct the models. Prior to the analysis, binary variables and ordinal categorical variables were appropriately assigned numerical values, while nominal categorical variables were entered into the models using dummy variable coding. The specific assignment schemes are presented in Table 3. Multicollinearity was assessed, and the variance inflation factor (VIF) for each predictor variable did not exceed 5.0, indicating no significant multicollinearity among the variables. The Durbin-Watson (DW) test values for the regression models were 1.936, 2.092, 1.875, and 1.940, respectively, all close to 2, suggesting that the residuals were independent.

**Table 3.** Coding of independent variables.

Variable	Coding
Age (years)	Raw value entered
Education Level	Junior college = 1; Bachelor’s Degree = 2; Master’s Degree and above = 3
Professional Title	Primary = 1; Intermediate = 2; Associate Senior = 3; Senior (Full)=4
Hospital type	Private = 0; Public = 1
Teaching hospital	No = 0; Yes = 1
Hospital level	Primary hospital (reference)
Tertiary hospital	Tertiary hospital = 1; Secondary hospital = 0; Primary hospital = 0
Secondary hospital	Tertiary hospital = 0; Secondary hospital = 1; Primary hospital = 0
Primary hospital	Tertiary hospital = 0; Secondary hospital = 0; Primary hospital = 1
Experience in Hemodialysis (Years)	≤5 = 1;6 ∼ 10 = 2;11 ∼ 15 = 3;≥16 = 4
Employment Type	Regular Staff (reference)
Contract Staff	Contract Staff = 1; Staff under Personnel Agency = 0; Dispatched Staff = 0
Staff under Personnel Agency	Contract Staff = 0; Staff under Personnel Agency = 1; Dispatched Staff = 0
Dispatched Staff	Contract Staff = 0; Staff under Personnel Agency = 0; Dispatched Staff = 1
Number of dialysis machines in the center	≤20 = 1;21 ∼ 40 = 2;41 ∼ 60 = 3;>60 = 4
Daily dialysis shifts	1 = 1; 2 = 2; 2 ∼ 3=3; 3 = 4; 3 ∼ 4=5
Conduct VTE risk assessment	No (reference)
Yes, assessment is mandatory	Yes, assessment is mandatory = 1; Yes, assessment is implemented but not mandatory = 0
Yes, assessment is implemented but not mandatory	Yes, assessment is mandatory = 0; Yes, assessment is implemented but not mandatory = 1
Awareness of VTE-related guidelines	Not familiar at all = 1; Slightly familiar = 2; Moderately familiar = 3; Somewhat familiar = 4; Very familiar = 5
Number of VTE-related training sessions attended in the past year	0 = 1; 1 ∼ 2=2; 3 ∼ 4=3; ≥5 = 4

Results of multiple stepwise linear regression analysis are presented in Table 4. For the total score, multivariate analysis identified significant predictors, including age (*β* = 0.165, *p* < 0.001), hospital type (*β* = 0.189, *p* < 0.001), daily dialysis shifts (*β* = 0.106, *p* = 0.021), mandatory implementation of VTE risk assessment (*β* = 0.162, *p* < 0.001), awareness of VTE-related guidelines (*β* = 0.226, *p* < 0.001), the number of VTE-related training sessions attended in the past year (*β* = 0.100, *p* = 0.023), and hospital tier. Notably, staff at secondary hospitals scored significantly lower in the total score compared to independent dialysis centers (*β* = −0.085, *p* = 0.04). The model’s adjusted R-squared was 0.264, explaining 26.4% of the variance.

**Table 4.** Results of multiple linear regression analysis on self-perceived knowledge, attitude and practice of VTE prevention among hemodialysis professionals (*N* = 474).

Model	Variable	*β*	*t*	95.0% CI	*p*
Total score	(Const)		14.008	(87.53,116.096)	<0.001***
	Age	0.165	3.772	(0.275,0.872)	<0.001***
	Hospital type	0.189	3.748	(5.369,17.204)	<0.001***
	Daily dialysis shifts	0.106	2.319	(0.445,5.389)	0.021*
	Hospital level (with independent dialysis centers as the reference)				
	Secondary hospital	−0.085	−2.062	(−17.562,−0.423)	0.04*
	Conduct VTE risk assessment (using the non-evaluated group as the reference)				
	Yes, assessment is mandatory	0.162	3.944	(5.05,15.078)	<0.001***
	Awareness of VTE-related guidelines	0.226	5.142	(4.723,10.566)	<0.001***
	Number of VTE-related training sessions attended in the past year	0.100	2.288	(0.565,7.44)	0.023*
self-perceived knowledge	(Const)		6.456	(17.98,33.716)	<0.001***
	Age	0.137	3.049	(0.101,0.468)	0.002**
	Education Level	0.095	2.016	(0.073,5.688)	0.044*
	Hospital type	0.238	4.883	(5.066,11.89)	<0.001***
	Hospital level (with independent dialysis centers as the reference)				
	Secondary hospital	−0.092	−2.226	(−10.913,−0.679)	0.026*
	Conduct VTE risk assessment (using the non-evaluated group as the reference)				
	Yes, assessment is mandatory	0.110	2.65	(1.054,7.098)	0.008**
	Awareness of VTE-related guidelines	0.191	4.328	(2.105,5.605)	<0.001***
	Number of VTE-related training sessions attended in the past year	0.130	2.93	(1.012,5.136)	0.004**
Attitude	(Const)		65.612	(59.543,63.219)	<0.001***
	Hospital level (with independent dialysis centers as the reference)				
	Tertiary hospital	0.144	3.084	(1.084,4.888)	0.002**
	Experience in Hemodialysis	0.174	3.64	(0.826,2.764)	<0.001***
	Employment Type (with the regular staff status as the reference)				
	Staff under Personnel Agency	−0.088	−1.983	(−20.789,−0.095)	0.048*
	Conduct VTE risk assessment (using the non-evaluated group as the reference)				
	Yes, assessment is mandatory	0.136	3.008	(0.989,4.717)	0.003**
Practice	(Const)		12.199	(19.295,26.704)	<0.001***
	Hospital type	0.099	2.074	(0.103,3.830)	0.039*
	Daily dialysis shifts	0.094	2.01	(0.019,1.703)	0.045*
	Conduct VTE risk assessment (using the non-evaluated group as the reference)				
	Yes, assessment is mandatory	0.309	5.969	(4.264,8.449)	<0.001***
	Yes, assessment is implemented but not mandatory	0.178	3.536	(1.467,5.137)	<0.001***
	Awareness of VTE-related guidelines	0.276	6.347	(2.141,4.061)	<0.001***

Note:*p < 0.05, **p < 0.01, ***p < 0.001

For self-perceived knowledge scores, significant predictors included age (*β* = 0.137, *p* = 0.002), education level (*β* = 0.095, *p* = 0.044), hospital type (*β* = 0.238, *p* < 0.001), and hospital tier. Notably, staff at secondary hospitals scored significantly lower in self-perceived knowledge compared to independent dialysis centers (*β*=-0.092, *p* = 0.026). Additionally, mandatory VTE risk assessment within the institution (*β* = 0.110, *p* = 0.008) compared to non-assessment groups, awareness of VTE-related guidelines (*β* = 0.191, *p* < 0.001), and the number of training sessions attended in the past year (*β* = 0.130, *p* = 0.004) were also significantly positively predictive of self-perceived knowledge scores. Key determinants of attitude scores included hospital tier and work experience. The model’s adjusted R-squared was 0.255, explaining 25.5% of the variance.

When comparing independent dialysis centers, working in tertiary hospitals was associated with higher attitude scores (*β* = 0.144, *p* = 0.002). More extensive hemodialysis experience (*β* = 0.174, *p* < 0.001) and mandatory VTE risk assessment within the institution (*β* = 0.136, *p* = 0.003) also showed significant positive effects on attitude. In contrast, when comparing formally employed staff, personnel hired through staffing agencies demonstrated significantly lower attitude scores (*β* = −0.088, *p* = 0.048). The model’s adjusted R-squared was 0.072, explaining 7.2% of the variance.

The primary associated factors for practice scores were associated with the assessment system and awareness. Mandatory VTE risk assessment was the strongest positive predictor (*β* = 0.309, *p* < 0.001), while non-mandatory but implemented assessments also showed a significant positive effect (*β* = 0.178, *p* < 0.001). Additionally, awareness of VTE-related guidelines (*β* = 0.276, *p* < 0.001), specific hospital characteristics (*β* = 0.099, *p* = 0.039), and more daily shifts (*β* = 0.094, *p* = 0.045) were significantly associated with better practice behaviors. The model’s adjusted R-squared was 0.208, explaining 20.8% of the variance.

## 4. Discussion

### 4.1. Hemodialysis professionals’ KAP of VTE prevention

This study investigated the KAP levels regarding VTE prevention in MHD patients among 474 hemodialysis healthcare professionals from multi-regional hospitals in China. The overall KAP was moderate (standardized score: 71.36 ± 12.06), indicating considerable room for improvement. The findings revealed a hierarchical pattern: attitude scores were the highest (82.31 ± 11.73), followed by practice (70.71 ± 16.76), while self-perceived knowledge scores were the lowest (62.51 ± 17.40). Our findings are consistent with a study from Turkey [21], which reported that healthcare workers had moderate levels of knowledge and positive attitudes; however, they contrast with a study from Guinea [22]. in which both knowledge and practice levels were low, possibly reflecting the impact of different healthcare resource levels. This result highlights the critical gap between guideline recommendations and clinical practice – a challenge consistently noted in the global literature on VTE prevention [23,24].

MHD patients exhibit chronic inflammation, vascular endothelial injury, coagulation dysfunction, and frequent vascular access use; these factors collectively predispose them to VTE [8–10]. These features distinguish VTE prevention in MHD patients from that in the general hospitalized population. Despite these elevated risks, current VTE prevention training for hemodialysis professionals predominantly emphasizes general VTE knowledge rather than dialysis-specific content. This directly contributes to the knowledge deficits observed in the present study, where scores on items requiring understanding of primary risk factors (e.g. antithrombin deficiency) and contraindications (e.g. cautious use of intermittent pneumatic compression in patients with lower-extremity atherosclerosis) were particularly low. Although attitude scores were high, practice scores remained moderate, further indicating that the translation of knowledge into proactive preventive behaviors is hindered by the lack of specialty-specific training and organizational support.

The specific self-perceived knowledge deficits identified – particularly regarding preventive measures and the application of risk assessment tools – highlight a targeted area for educational intervention. This finding is consistent with reports from other countries, where healthcare professionals often demonstrate familiarity with VTE risks but lack detailed self-perceived knowledge of standardized prophylaxis protocols [25]. The high scores on core self-perceived knowledge items (e.g. mechanisms, risk factors) suggest that current training may effectively convey theoretical foundations but fall short in translating them into practical, tool-based competency. Similarly, the practice dimension revealed strengths in reactive care (e.g. emergency response) but weaknesses in systematic, proactive measures such as routine risk assessment and monitoring of laboratory parameters. This discrepancy between theoretical recognition and procedural execution has been documented in previous studies and points to systemic rather than individual barriers, including time constraints, workflow integration issues, and insufficient training on specific tools [21,26].

### 4.2. Factors associated with KAP among hemodialysis professionals regarding VTE prevention

The correlation coefficients between the total score and each dimension were very high (0.711–0.875), which can be partially attributed to mathematical overlap, as the total score is the sum of the three dimension scores. The three KAP dimensions (knowledge-attitude: *r* = 0.408; knowledge-practice: *r* = 0.424; attitude-practice: *r* = 0.389; all *p* < 0.001) reinforce the mutually reinforcing theoretical framework. These values are quantitatively comparable to those reported in studies on nurse-led VTE prevention in other patient populations [27]. This consistency supports the underlying KAP model that enhanced self-perceived knowledge fosters positive attitudes, which in turn facilitate improved practices. The association suggests that interventions aiming to change practice must simultaneously address both cognitive (self-perceived knowledge) and motivational (attitude) components, rather than focusing on one alone.

#### 4.2.1. Demographic and occupational factors

The multivariate analysis identified key demographic and occupational factors associated with KAP levels, such as younger age, shorter work experience, and lower professional titles. These groups represent primary targets for foundational training. This finding resonates with international studies indicating that clinical experience and advanced training are positively correlated with better VTE prevention practices [28]. In the present study, private and independent dialysis centers accounted for 68.57% and 60.97% of the sample, respectively, and their healthcare professionals had significantly lower KAP scores than those in public tertiary hospitals. This phenomenon is closely related to the aforementioned individual factors: in resource-limited institutions, younger and lower-title personnel find it even more difficult to access systematic training and institutional support, thus their knowledge-practice gap is more pronounced. Therefore, interventions targeting these institutions should consider both individual capacity building and organizational environment improvement.

#### 4.2.2. Organizational environmental factors

Hospital type and level had a significant impact on KAP scores, pointing to broader structural determinants. In this study, healthcare professionals in public hospitals and tertiary hospitals had significantly higher KAP scores (*p* < 0.001), whereas those in private and independent dialysis centers scored lower. This aligns with research emphasizing the role of organizational context and quality management systems in enabling consistent preventive care [29]. Specifically, economically developed regions and tertiary hospitals generally have more established VTE prevention management systems, clear assessment protocols, and rigorous supervision and evaluation mechanisms, whereas primary-level and private institutions have lower policy implementation levels, leading to less standardized practice execution. Workload is an important organizational factor constraining practice levels [30]. In this study, 64.14% of healthcare professionals worked more than 40 h per week, and this high workload made it difficult to conduct dynamic risk assessments during busy shifts, which further hampers the standardized implementation of preventive measures. Moreover, most institutions lack mandatory requirements for routine VTE risk reassessment. This organizational absence of mandate directly contrasts with the finding that ‘mandatory assessment is the strongest predictor’, highlighting the necessity of establishing a routine risk reassessment process.

#### 4.2.3. Institutional and training factors

Whether a department conducts VTE risk assessment is a common influencing factor for the total score and all dimensions (*p* < 0.05). In departments where VTE risk assessment is mandatory, healthcare workers’ KAP levels are significantly higher than those in departments without mandatory assessment or without any assessment, suggesting that institutionalized management plays an important role in promoting the implementation of VTE preventive measures. Previous studies have indicated that integrating risk assessment into routine nursing processes and establishing standardized management requirements are important drivers for transforming healthcare workers’ knowledge and attitudes into actual practice [31]. When VTE risk assessment is incorporated into daily work routines and required to be performed in a standardized manner, it can strengthen healthcare workers’ sense of responsibility, enhance their awareness of the importance of VTE prevention, and thereby promote the application of theoretical knowledge in clinical practice, gradually forming normalized and standardized preventive behaviors. Conversely, in the absence of clear institutional mandates and monitoring mechanisms, VTE preventive measures tend to be weakened, their priority is lowered, and they become difficult to sustain over the long term, thus compromising prevention outcomes.

The results of this study showed that the number of VTE-related training sessions attended in the past year had statistically significant differences in the total KAP score, knowledge dimension score, and practice dimension score among healthcare workers (*p* < 0.001). As the number of training sessions increased, the total KAP score and dimension scores of healthcare workers showed an upward trend, with the highest scores observed among those who attended 3–4 sessions, suggesting that systematic and continuous training helps improve healthcare workers’ mastery of VTE prevention-related knowledge and facilitates the implementation of preventive measures. Meanwhile, this study also found that awareness of VTE-related guidelines was an important factor influencing the total KAP score and knowledge dimension score (*p* < 0.001). Healthcare workers with a higher level of awareness of VTE-related guidelines had significantly higher KAP scores than those unaware of the guidelines, suggesting that guideline learning plays an important role in enhancing healthcare workers’ VTE prevention capabilities. Previous studies have shown that continuous professional training can effectively improve healthcare workers’ compliance with VTE risk identification and implementation of preventive measures, serving as an important pathway to promote practice implementation [32]. Therefore, the VTE training system should be further improved by implementing tiered and categorized training based on different roles, years of experience, and specialty characteristics, while strengthening the integration of guideline interpretation with practical operations, so as to continuously enhance the VTE prevention capabilities and behavioral performance levels of hemodialysis healthcare workers.

In conclusion, this study revealed a moderate overall KAP level with identifiable gaps in self-perceived knowledge and procedural practice among hemodialysis professionals regarding VTE prevention in MHD patients. The correlations among dimensions confirmed the interrelated nature of KAP. To enhance prevention efficacy, at the institutional level: (1) establish routine VTE risk assessment supervision mechanisms, with designated personnel monitoring completion rates and providing regular feedback; (2) integrate VTE assessment adherence into performance evaluations; (3) develop standardized workflows specifying assessment frequency, validated tools adapted for the dialysis population, and corresponding preventive actions; (4) implement regular audit-feedback systems with monthly compliance reviews. At the training level: (1) shift from purely theoretical instruction to simulation-based training using hemodialysis-relevant case scenarios, with emphasis on dynamic risk assessment skills; (2) develop MHD-specific training modules addressing the unique VTE risks of this population. At the policy level: (1) explore strategies to optimize staffing ratios, such as designating specialized nurse coordinators for vascular access and thrombosis prevention, to allocate dedicated time for preventive activities.

### 4.3. Limitations

There are several limitations to this study. First, the knowledge dimension was measured based on participants’ subjective self-perceived mastery rather than objective assessment, which may not accurately reflect their actual professional competence. Second, the study used convenience sampling and was restricted to a disproportionately female sample, introducing sampling bias that limited the representativeness and generalizability of the conclusions. Third, owing to its cross-sectional design, this study could only identify correlational relationships among variables; no causal or temporal inferences could be drawn. Fourth, all data were collected *via* online self-administered questionnaires, which are inherently susceptible to recall bias and social desirability bias. Fifth, the standardized scoring method may lead to slight overestimation of the overall KAP level. In addition, the use of stepwise regression carries inherent limitations such as potential overfitting.

## 5. Conclusion

In conclusion, this multicenter cross-sectional study of 474 hemodialysis professionals revealed moderate levels of VTE prevention self-perceived knowledge and practice, and positive attitudes. Key associated factors included institutional VTE assessment protocols, guideline awareness, training frequency, and hospital characteristics. Notably, self-perceived knowledge and practice were more influenced by systemic factors, while attitudes were influenced by individual and organizational factors. Future research should employ longitudinal designs to establish causal relationships, develop and test targeted interventions, and explore unmeasured factors contributing to the KAP-practice gap. Implementation science approaches are needed to translate evidence into sustainable improvements in VTE prevention for hemodialysis patients.

## Supplementary Material

Supplemental Material

## Data Availability

The datasets used and/or analyzed during the current study are available from the corresponding author on reasonable request.
