# Supplementary material for: Hemodialysis professionals’ knowledge, attitudes, and practices toward venous thrombosis prevention: a multicenter survey
Source: Ren Fail. 2026 Sep 14;48(1):2707036. doi: 10.1080/0886022X.2026.2707036 (PMC13577280; doi:10.1080/0886022X.2026.2707036)
Supplement: Supplementary file 1 [file IRNF_A_2707036_SM7384.pdf]

Questionnaire on self-perceived knowledge, Attitudes, and Practice of VTE Prevention among Hemodialysis Professionals

Part I: Self-perceived knowledge of VTE prevention among hemodialysis professionals

1. Venous thromboembolism (VTE) refers to abnormal blood clotting within a vein, leading to partial or complete lumen obstruction.
   ☐ Completely unaware ☐ Partially aware ☐ Aware ☐ Familiar ☐ Master
2. Venous thromboembolism (VTE) includes deep vein thrombosis (DVT) and pulmonary thromboembolism (PTE).
   ☐ Completely unaware ☐ Partially aware ☐ Aware ☐ Familiar ☐ Master
3. The main mechanisms of VTE are hypercoagulability, venous stasis, and vessel wall injury.
   ☐ Completely unaware ☐ Partially aware ☐ Aware ☐ Familiar ☐ Master
4. Antithrombin deficiency, hyperhomocysteinemia, congenital dysfibrinogenemia, etc., are primary risk factors for deep vein thrombosis.
   ☐ Completely unaware ☐ Partially aware ☐ Aware ☐ Familiar ☐ Master
5. Advanced age, prolonged immobilization, malignancy, surgery, heart failure, history of VTE, etc., are risk factors for VTE.
   ☐ Completely unaware ☐ Partially aware ☐ Aware ☐ Familiar ☐ Master
6. The longer the central venous catheter is indwelling, especially femoral vein catheterization, the higher the risk of VTE for the patient.
   ☐ Completely unaware ☐ Partially aware ☐ Aware ☐ Familiar ☐ Master
7. Fluid volume changes before and after hemodialysis in MHD patients affect hemodynamics, thereby influencing VTE risk.
   ☐ Completely unaware ☐ Partially aware ☐ Aware ☐ Familiar ☐ Master
8. Hemodialysis patients have a higher risk of VTE compared with the general population, due to the use of extracorporeal blood circuits and vascular access.
   ☐ Completely unaware ☐ Partially aware ☐ Aware ☐ Familiar ☐ Master
9. MHD patients with underlying diseases such as nephrotic syndrome, diabetic nephropathy, systemic lupus erythematosus, antiphospholipid syndrome, etc., have an elevated risk of VTE.
   ☐ Completely unaware ☐ Partially aware ☐ Aware ☐ Familiar ☐ Master
10. The main clinical manifestations of acute PTE include dyspnea, tachypnea, cyanosis, cough, hemoptysis, decreased oxygen saturation, etc.
    ☐ Completely unaware ☐ Partially aware ☐ Aware ☐ Familiar ☐ Master
11. Clinical manifestations of acute lower extremity DVT include limb swelling, pain, increased skin temperature, etc.
    ☐ Completely unaware ☐ Partially aware ☐ Aware ☐ Familiar ☐ Master
12. Regular monitoring of patients' coagulation function, plasma D-dimer, thromboelastography, etc., is helpful for VTE prevention.
    ☐ Completely unaware ☐ Partially aware ☐ Aware ☐ Familiar ☐ Master
13. Color Doppler ultrasonography is the first-line method for diagnosing DVT.
    ☐ Completely unaware ☐ Partially aware ☐ Aware ☐ Familiar ☐ Master
14. CT pulmonary angiography (CTPA) is the first-line method for diagnosing PTE.
    ☐ Completely unaware ☐ Partially aware ☐ Aware ☐ Familiar ☐ Master

15. VTE prevention measures include basic prevention, mechanical prevention, and pharmacological prevention.
☐ Completely unaware ☐ Partially aware ☐ Aware ☐ Familiar ☐ Master
16. Ankle pump exercise is a basic VTE prevention measure.
☐ Completely unaware ☐ Partially aware ☐ Aware ☐ Familiar ☐ Master
17. Mechanical VTE prevention includes graduated compression stockings (GCS), intermittent pneumatic compression devices (IPC), and venous foot pumps (VFPs).
☐ Completely unaware ☐ Partially aware ☐ Aware ☐ Familiar ☐ Master
18. MHD patients with concurrent lower extremity arterial disease should use intermittent pneumatic compression devices with caution.
☐ Completely unaware ☐ Partially aware ☐ Aware ☐ Familiar ☐ Master
19. For patients at moderate-to-high VTE risk with low bleeding risk, pharmacological prevention alone or in combination with mechanical prevention is recommended as first-line treatment.
☐ Completely unaware ☐ Partially aware ☐ Aware ☐ Familiar ☐ Master

Part II: Attitudes toward VTE prevention among hemodialysis professionals

1. I believe that VTE risk should be dynamically assessed based on the patient's vascular access type, dialysis modality, anticoagulation regimen, etc.
☐ Strongly disagree ☐ Disagree ☐ Uncertain ☐ Agree ☐ Strongly agree
2. I believe that attention should be paid to the patient's vascular access type and its use status.
☐ Strongly disagree ☐ Disagree ☐ Uncertain ☐ Agree ☐ Strongly agree
3. I believe that attention should be paid to the patient's VTE-related examination and laboratory results.
☐ Strongly disagree ☐ Disagree ☐ Uncertain ☐ Agree ☐ Strongly agree
4. I believe that patients assessed as high VTE risk should receive anticoagulation education and lifestyle guidance.
☐ Strongly disagree ☐ Disagree ☐ Uncertain ☐ Agree ☐ Strongly agree
5. I believe that VTE prevention efforts should be strengthened for MHD patients who are elderly or have underlying diseases such as diabetes mellitus.
☐ Strongly disagree ☐ Disagree ☐ Uncertain ☐ Agree ☐ Strongly agree
6. Before initiating anticoagulation therapy, I believe that a dynamic assessment of both bleeding risk and VTE risk should be performed for the patient.
☐ Strongly disagree ☐ Disagree ☐ Uncertain ☐ Agree ☐ Strongly agree
7. I believe that mastering the clinical manifestations of VTE is essential for early identification and prevention of VTE.
☐ Strongly disagree ☐ Disagree ☐ Uncertain ☐ Agree ☐ Strongly agree
8. I believe that caution is needed when managing patients with catheter dysfunction, especially when femoral vein catheter thrombosis is suspected.
☐ Strongly disagree ☐ Disagree ☐ Uncertain ☐ Agree ☐ Strongly agree
9. When VTE prevention measures conflict with the need for urgent dialysis treatment, I believe that the risks of both should be balanced, and an individualized approach should be adopted.

☐ Strongly disagree ☐ Disagree ☐ Uncertain ☐ Agree ☐ Strongly agree

10. I believe that healthcare providers should jointly develop a multidisciplinary VTE prevention plan for MHD patients.
    ☐ Strongly disagree ☐ Disagree ☐ Uncertain ☐ Agree ☐ Strongly agree
11. I believe that VTE-related training for healthcare workers is needed at the departmental level.
    ☐ Strongly disagree ☐ Disagree ☐ Uncertain ☐ Agree ☐ Strongly agree
12. I believe that regular learning of new advances in VTE prevention and treatment is crucial for improving patient care quality.
    ☐ Strongly disagree ☐ Disagree ☐ Uncertain ☐ Agree ☐ Strongly agree
13. I believe that family members of patients at high VTE risk should be trained in the emergency management of VTE (e.g., hemoptysis, sudden chest pain, etc.).
    ☐ Strongly disagree ☐ Disagree ☐ Uncertain ☐ Agree ☐ Strongly agree
14. I believe that patients at moderate-to-high VTE risk should be followed up.
    ☐ Strongly disagree ☐ Disagree ☐ Uncertain ☐ Agree ☐ Strongly agree
15. I believe that I should master the methods for assessing both bleeding and VTE risks.
    ☐ Strongly disagree ☐ Disagree ☐ Uncertain ☐ Agree ☐ Strongly agree
16. I believe that VTE risk assessment should be a routine check for high-risk populations before each dialysis session.
    ☐ Strongly disagree ☐ Disagree ☐ Uncertain ☐ Agree ☐ Strongly agree

Part III: Practices of VTE prevention among hemodialysis professionals

1. I use risk assessment tools to perform VTE risk assessment for patients.
   ☐ Never ☐ Rarely ☐ Sometimes ☐ Often ☐ Always
2. When changing the hemodialysis access type, dialysis modality, etc., I perform dynamic VTE reassessment and provide preventive measures.
   ☐ Never ☐ Rarely ☐ Sometimes ☐ Often ☐ Always
3. I perform preventive care for catheter-related thrombosis in patients with indwelling hemodialysis catheters.
   ☐ Never ☐ Rarely ☐ Sometimes ☐ Often ☐ Always
4. I adjust VTE prevention measures according to the patient's dialysis modality and vascular access type.
   ☐ Never ☐ Rarely ☐ Sometimes ☐ Often ☐ Always
5. I promptly follow up on patients' VTE-related examinations and laboratory indicators.
   ☐ Never ☐ Rarely ☐ Sometimes ☐ Often ☐ Always
6. When a patient shows suspected VTE symptoms, I immediately report it to the physician and provide initial management.
   ☐ Never ☐ Rarely ☐ Sometimes ☐ Often ☐ Always
7. I instruct bedridden patients to perform ankle pump exercises correctly to prevent VTE.
   ☐ Never ☐ Rarely ☐ Sometimes ☐ Often ☐ Always
8. I implement appropriate preventive measures and health education for patients assessed at different risk levels.
   ☐ Never ☐ Rarely ☐ Sometimes ☐ Often ☐ Always

9. I conduct regular follow-up for patients assessed as moderate-to-high VTE risk and those diagnosed with VTE, and complete the follow-up forms.
   ☐ Never ☐ Rarely ☐ Sometimes ☐ Often ☐ Always
10. I update my knowledge and skills related to VTE prevention by attending training sessions, reviewing literature, etc.
   ☐ Never ☐ Rarely ☐ Sometimes ☐ Often ☐ Always
11. After a VTE event occurs in an MHD patient within my department, I participate in case discussions and summarize deficiencies in preventive measures.
   ☐ Never ☐ Rarely ☐ Sometimes ☐ Often ☐ Always
